# Do qualifications matter? A qualitative study of how villagers decide their health care providers in a developing economy

**DOI:** 10.1371/journal.pone.0220316

**Published:** 2019-08-01

**Authors:** Gopesh Anand, Dilip Chhajed, Shailja Shah, Salla Atkins, Vishal Diwan

**Affiliations:** 1 Department of Business Administration, Gies College of Business, University of Illinois at Urbana-Champaign, Illinois, United States of America; 2 Indian Institute of Public Health, Gandhi Nagar, India; 3 New Social Research and Health Sciences, Faculty of Social Sciences, University of Tampere, Tampere, Finland; 4 Department of Public Health Sciences, Karolinska Institutet, Stockholm, Sweden; 5 Department of Public Health and Environment, R.D. Gardi Medical College, Ujjain, India; 6 International Centre for Health Research, Ujjain Charitable Trust Hospital and Research Centre, Ujjain, India; Yale University Yale School of Public Health, UNITED STATES

## Abstract

**Introduction:**

The National Rural Health Mission (NRHM) was launched in India in 2005 to address the health needs of under-served populations in rural areas, and to support universal access to care. Despite this initiative, unaccredited informal providers (IPs) often remain patients’ first point of contact, which has led to inconsistencies in treatment, and has compromised the quality of care.

**Aim:**

To explore the factors that influence patients’ decisions about healthcare providers in rural areas of central India.

**Methods:**

Nine focus group discussions (FGDs) were held in nine villages in central India. Framework analysis using an inductive approach was used to analyse the data.

**Results:**

The crosscutting theme across the discussions was not choice but need—the need for affordable and accessible health care regardless of the provider’s qualification. Results highlighted that IPs play a pivotal role in villagers’ lives. Formal healthcare services were accessed infrequently, and mainly when a condition was judged severe or possibly even fatal. Even then, affordability was carefully weighed. Villagers’ distance from formal providers contributed to high cost and low preference of formal providers. When opting for IPs, familiarity and trust were more important to villagers than qualifications. IPs have operated in rural communities in India for a long time and have adapted their services to meet the needs, preferences, social norms, and economic conditions of villagers.

**Conclusion:**

IPs have captured a niche and are often the first contact point in rural settings even when patients ultimately are diagnosed and treated by trained doctors. Merely tackling the undersupply of qualified doctors is not effective or sufficient to impact on the rural healthcare system: the strong and prevalent influence of IPs needs to be addressed also.

## Introduction

Universal health coverage (UHC) was recognized as a goal for healthcare systems globally in 1978 [1] and was further affirmed by the sustainable development goals [2]. UHC implies equitable access to key healthcare services, including promotive, preventive, curative and rehabilitative health interventions at affordable cost to all [3]. In India, The National Rural Health Mission was launched in 2005 to support this, and improve access to public health care services, especially for the underprivileged population [4]. The three-tier system of public health care services consists of sub-centres, primary health centres (PHCs), and community health centres (CHCs). There are approximately 23,458 PHCs and 4,276 CHCs in the country. However, human resources in the public healthcare sector are scarce: 18.8% of PHC and 51.8% of CHC doctor positions remain unfilled [5]. Approximately 74% of medical graduates offer services to 28% of the country’s population, mostly comprising affluent families in urban settings [6]. Qualified or formal providers (FPs), those recognized by a government-affiliated regulatory body, are often unwilling to practice in rural health centres [7]. Therefore, despite the National Rural Health Mission, the public health system in India has consistently fallen short of almost all its goals, including quality of care, and physical accessibility of services, especially for rural residents for whom most care is provided by non-qualified persons [4,8]. Those formal health services that are available in rural areas are reportedly nonresponsive to people’s needs [9].

Given the challenges listed above, informal practitioners (IPs), practitioners who have some level of informal training but are not registered as healthcare workers nor formally accredited [10], have become the principal health care providers in rural areas of India [11]. IPs include traditional birth attendants, spiritual leaders, unqualified doctors and unqualified doctors [9]. IPs are different from community health workers (CHWs) and accredited social health activists (ASHA) where the latter interacts with the rural population through a government supported platform and acts as liaison between individual and government health facilities. Though it is widely known that IPs are the first point of contact for many rural patients, there is no formal mechanism of getting information about the exact number of IPs in India, nor is there a database of how many providers are currently active in different parts of India. However, according to available reports, IPs constitute approximately more than half of all active providers in rural India [12] and they are often the first point of contact when residents have health problems [5]. Despite being legally prohibited from practising, a census of providers in rural Madhya Pradesh revealed the existence of 12 times more IPs than FPs [13]. As much as 77% of rural health care services are provided by IPs in the central Indian state of Madhya Pradesh [14]. This is a problem for population health as the untrained and entrepreneurial IPs cannot provide the same quality of health services as FPs [15]. Studies reporting on the training of IPs to improve their provision of care suggests that training can improve rates of correct case management but has only limited impact on correct medicine prescription [13].

An argument could be made that IPs proliferate in rural areas because there is demand for their services. However, there are few investigations of why villagers, who are often underprivileged, opt for IPs. This information is key to designing and improving the rural health services and to inform health care delivery for underserved populations [16] in India and other low-and middle-income settings. This paper aims to understand what influences decisions about choosing healthcare providers (IP or FP) among rural residents in India.

## Methods

This is a qualitative study using focus group discussions (FGDs).

### Study setting

This study was conducted in the Ujjain district of Madhya Pradesh state, India. Ujjain is one of 51 administrative districts and has a population of 1.9 million people [17]. The human development index (HDI) of Madhya Pradesh is 0.557, and it is ranked 20^th^ among 29 states and 7 union territories in the country [18]. Approximately 49% of Ujjain’s population live below the poverty line [19], as compared to the Indian average of 21.2%. The sex ratio in the district is 968 females per 1,000 males [20]; the maternal mortality rate is 176 per 100,000 live births, and the infant mortality rate is 54 per 100,000 live births [21], which is worse than the country average. The population density is 326 people per square kilometre. The literacy rate is 72.3% [17]. The health system in Ujjain is made up of the public and private sector. In total, 56.0% of all health care providers in Ujjain are IPs, with more IPs present in rural areas [18].

### Participants, sampling and data collection

Purposive sampling was used for selecting the site and the participants. The study was conducted in rural demographic surveillance site (DSS) of R D Gardi Medical College, Ujjain. R D Gardi Medical College has been providing health care services to this area for more than 15 years through its rural health centre and also through its network of village health workers. The medical college has built a rapport with the community through its health services programs.

Initially 12 villages were randomly selected out of sixty villages in the DSS to carry out the FGDs. We sought to understand the situation of deciding about health providers in a variety of settings, and therefore conducted FGDs in different villages in 2014. We randomly selected 20 households in each village. Local health workers visited the chosen households to inform about the study and invited one adult household member for the FGD. Participants were included if they, or a family member, had visited a health care practitioner at least once in the 30 days preceding the FGD date.

We used a piloted semi-structured topic guide to conduct the FGDs. It was prepared based on a review of literature, and covered the domains of health-seeking behaviour, approaching IPs, factors influencing patient choices, and IPs’ attributes (Appendix A). The FGDs were carried out in a regional dialect in which both the participants and the data collectors were well versed. Each FGD consisted of between seven and 12 participants. The sample size was informed by data saturation, as we conducted analysis during data collection. After the ninth FGD we found that no new themes were emerging, and closed sampling. Two FGDs were conducted by female moderator with groups of women; seven were conducted by male moderator (VD) with groups of men. Each FGD lasted between 45–60 minutes and was audio-taped (see Table 1 for participant characteristics). The researchers also took detailed notes to facilitate transcription of the recordings. The participants were given light refreshment after the FGDs. No other incentives were offered to study participants.

**Table 1:** Characteristics of FGD participants.

FGD no	Type	No of Participants	Age Range (years)	Number of years of schooling (Mean years ± SD)
1	Male	8	22–28	8.1±2.9
2	Male	10	32–41	6.1±3.9
3	Male	12	46–65	5.0±4.2
4	Female	8	35–50	2.8±3.2
5	Male	7	22–29	7.7±2.0
6	Male	10	34–50	9.9±2.3
7	Male	10	18–33	7.5±3.7
8	Male	8	45–66	4.5±4.0
9	Female	8	20–29	4.5±4.0

### Data analysis

Transcription was done in Hindi and the content was translated into English by VD and research assistants. Data analysis was conducted inductively, concurrently with FGDs and sampling continued until data saturation. We used framework analysis to analyse the data [22]. The approach involves reading and familiarising with the data, coding, creating a framework from these codes and finally charting the data onto the framework. Two authors (GA and VD) analysed the data independently, and differences were resolved through discussion. The authors then presented the data to the rest of the research team for agreement and refinement where necessary.

### Ethical considerations

The study was approved by ethics committee of R. D. Gardi Medical College, Ujjain, India (reference number 351, December 4, 2013). Written informed consent was obtained from all participants included in the study prior to the start of the FGDs. Permission was also sought from participants for recording the FGDs.

## Results

The main, crosscutting theme across the interviews was not choice, but need–the need for affordable and accessible healthcare, regardless of the provider’s qualifications. We expand on this theme below.

### Ubiquitously available informal providers ease access to healthcare

IPs are more readily accessible in rural areas than qualified doctors. Some IPs had worked or had been working for qualified doctors in the urban areas, and according to participants they practiced relying on their gained knowledge and experience. While FPs were available only during the day, IPs were also available at night:

*“We have a compounder* [a person who mixes medicines] *here who goes to Ghosla in day time*. *At night*, *we take medicine from him”*

*(FGD2*, *Male 4)*

There were far more IPs in the villages when compared with FPs. Some pharmacists acted as IPs, dispensing medication for ailments, and villagers also had access to traditional Ayurveda providers. However, in these rural villages qualified doctors and/or health facilities either did not exist at all or were perceived by participants as inadequate for their needs. For example, the provider’s gender was important for particularly women participants–they noted their preference for, and a shortage of, qualified female doctors in rural areas.

Participants also spoke of not visiting formal providers because they could not get there–there was insufficient transport available to travel outside the village and the travel would take a lot of time. This was closely linked to the overall issue of cost and affordability, described below. IPs tended to practice inside the villages, where qualified doctors were reluctant to open their clinics. Thus, for most villagers, the nearest health provider was an IP. Sometimes, IPs were also willing to make house calls, easing access barriers further:

*“Even if there is some emergency at night*, *they [local IPS] will readily wake up and treat the patient*. *If we take the patient to their place*, *they would treat and if we call them home*, *they would come home and treat”*

*(FGD 1*, *Male 3)*

However, even in those villages where government and charitable hospitals were available within reasonable distance, the participants reported avoiding going there because they found the prospect of having to deal with non-cooperative government hospital administrators unpleasant. Some participants also reflected on the importance of connections to receive good treatment. Reflecting on the scarcity of qualified doctors at tertiary care government hospitals, respondents also reported that such facilities had long waiting times, which was not convenient for them. Several respondents also said that in their opinion, doctors at charitable hospitals, such as those at the medical college, spend too much time in making a diagnosis, whereas IPs were ready to give treatment after a shorter period of investigation. IPs were therefore perceived a better choice, as they mostly lived close to the patients, were available more quickly, offered flexible hours, and had a readily available stock of medicines. These factors were seen as speeding up interactions to receive treatment.

### IPs are more affordable and offer flexible payment—Easing the burden on low income patients and increasing access

Affordability was a key issue behind participants’ decisions about different providers. While IPs readily worked on credit or even on a barter system, which was convenient to villagers with low incomes, qualified doctors required upfront payments even before they saw patients:

“*One of the other reasons for choosing local doctor [IPS] is financial problems because in villages we don’t always have money*. *We are all depended on agriculture and there is no regular income*. *So*, *for primary treatment*, *we opt for local doctors only*.”

*(FGD 2*, *Male 8)*

“We don’t have ready cash for any emergency… We are thankful to the village doctor who give us credit for 6 months.”

*(FGD5*, *Male1)*

In addition to flexible payment, credit, and bartering, the simple cost of services was a barrier to using FPs’ services:

*“Here the money charged is also very less [sic]*. *Here we can complete the treatment in Rupees 200 and in city we have to start with doctor’s fees of at least Rupees 200* [author note, 80 rupees = US$1.00].”

*(FGD 3*, *Male 6)*

*“We all think about this first*. *If we get good treatment by spending less money than that would be best*. *Some doctors charge Rupees 500 for just touching the patient’s hand*. *If we have two doctors and one is charging Rupees 50 and the other one is charging Rupees100 and both of them are curing patients*, *then everyone would go to the cheap doctor only.”*

*(FGD 9*, *Male 1)*

IPs provided low cost care and in addition offered a complete package–they provided apparently free diagnosis and charged only for medications. In contrast to FPs, IPs readily offered credit to their patients for consultation fees and medications. Sometimes, they even accepted produce in lieu of payment. The seasonal nature of work for villagers, many of whom relied on agriculture and farming, meant that they sometimes had little cash readily available for healthcare. This was an advantage to IPs: their charges were low when compared to those of FPs. Combined with the difficulty in reaching FPs and their cost, IPs seemed to be more affordable to the participants. Cost was also linked to access and the location of FPs: going to a city to access FPs could involve the additional expense for public transportation fare for at least two people:

*“It is nearly Rupees 30 by bus but if the patient is serious(ly ill) [sic]than we have to hire a vehicle which would cost us around Rupees 800*. *So*, *people think it is better to get treated here only.”*

*(FGD 9*, *Male 3)*

In addition to actual transport costs, visiting a FP in the city could take all day, which added the opportunity cost of lost time to affordability. A patient who went to a city for treatment not only had to find a way to reach the city and locate a suitable doctor, but once she or he reached the doctor’s office, had to wait in line, sometimes for several hours or even overnight, before the doctor could see him/her. For FPs, in addition, payment was due before the consultation, after which the patient had to pay for medicine at a pharmacy. The FP might also order laboratory investigations which require additional time before the treatment can begin. In contrast to this, the IPs in the village were close by, see the patient quickly, and give medicine immediately.

According to participants, patients escalated care-seeking to the next level–going to urban centres to FPs–after first trying treatments from local IPs. They went to different urban centres based on their perception of the doctors there, and sometimes also based on reference provided by the local IPs. Occasionally the local IPs even helped with transportation to urban centres to visit FPs when they (IPs) were not able to address the ailment.

*“The first reason for not going to Ujjain is to save time*. *When will we go to Ujjain and come back from there*, *it approximately takes 3 hours*. *In Ghosla we get immediate relief*. *We can reach Ghosla in 20 minutes and they start giving treatment for the illness.*

*(FGD 4*, *Male1)*

### Trust and reputation, social and cultural factors trumped qualifications—Except in emergencies

The respondents seemed to be aware of the qualifications that IPs had in comparison with FPs. However, participants indicated that they generally chose to go to FPs in the urban areas only after first trying treatment from local IPs.

*“For big diseases like heart problem*, *complications in fever*, *motiabind (cataract)*, *typhoid etc*. *we go to Ujjain*. *If the local doctors are not able to treat*, *then only we go to Ujjain”*

*(FGD 6*, *Male 2)*

Participants stated that emergencies were situations that resulted in a preference of FPs–an important condition, potentially life threatening, warranted more skilled care. Therefore, respondents reported at times going directly to the city, without trying IPs first.

Overall, word of mouth appeared to play a major role when selecting of specific IPs or FPs, and when choosing between the two options. It seemed from the groups that while some people were aware of the need for qualifications, others deliberately ignored the notion, and based their choice of provider on trust and reputation alone.

According to the participants, an IP was usually also acquainted with the patient. Some respondents did not even appear to be aware at all of the options available to them including free charitable clinics, rather trusting those IPs that were known to them and their families.

Trust and familiarity therefore played an important role in the choice of treatment that respondents sought out, closely related to social and cultural norms.

*“Yes*, *even if a particular doctor doesn’t have a degree*, *if we have faith on his treatment*, *we will go to him”*

*(FGD 8*, *Female 5)*

As the participants had limited knowledge of the required treatment contents and standards, beyond what the providers themselves described, they perceived diagnostic procedures initiated by FPs as a waste of time:

*“Nowadays they charge Rupees 300 and nothing less than that for just examining you*. *After that they would ask you to do many tests and to bring the report*. *Then only they would start the treatment*. *In village we will go to the doctor and he will directly give you tablet*, *and injection and you would be fine.”*

*(FGD 2*, *Male 9)*

However, when the medicine following a diagnostic procedure appeared to cure a patient, participants reported that the patients and their families appreciated the use of the procedure.

In addition to trust and familiarity with a provider, the participants reported that the decision to consult a particular healthcare provider (IP or FP) was usually made for the entire household by one family member, usually a patriarch.

“My husband only takes the decision to go to the doctor.”

*(FGD 7*, *female 2)*

“My grandfather decides for the family as he is the eldest in the family.”

*(FGD 7*, *female 5)*

## Discussion

This study identified factors that contribute to patients’ decisions about selecting IPs or FPs in rural areas in central India. We found that even after the implementation of the National Rural Health Mission in 2005, the reasons for rural patients preferring IPs remain similar to the ones identified in Rhode and Viswanathan’s [23] study, carried out nearly 25 years ago: the provision of fast, affordable treatment and flexible treatment options, and patients’ belief systems. Our study also confirms that IPs continue as a notable source of health care for rural residents [5,23–25]. A study of IPs in two districts in India reported that ‘doorstep’ services [5] were offered by mobile IPs who travelled from house to house. Similarly, we observed that some IPs provided house calls and also accompanied patients to formal health care providers when needed, which increased peoples’ trust in them. Gautham et al. [5] found that IPs fill the demand for primary curative care, which the public system does not satisfy, and are thus often the de facto first-level access points; our results suggest that this phenomenon persists.

Across our FGDs, respondents noted that IPs play a pivotal role in providing health care services and the first point of care in their villages. The NHM, deployed in 2005, had envisioned trained accredited social health activists (ASHAs) to be the first contact point for health-related demands in the communities [26]. However, according to our observations, the first contact point remains the IPs. These IPs treat minor illnesses and, if they are unable to treat an illness, also recommend formal doctors. We found that villagers’ preferences for utilising health care services were not static, but were case by case dependent on the accessibility, affordability, and cultural acceptability of the provided services. Our findings also confirm the results of a similar study in West Bengal, where the reasons for approaching IPs were proximity and round-the-clock availability [10]. Improving the public health system and to increase use of FPs, therefore requires revising and amending the existing three-tier system [5] to become more responsive to patient needs and preferences.

We found that most decisions to approach FPs or IPs were based on trust and familiarity. People’s preferences were dictated by their cultural values and their trust in IPs is accompanied by a distrust of the formal sector [10]. Respondents expressed general mistrust in government hospitals and believed that connections with influential people were necessary to be well treated there. There is some evidence that a lack of investment in rural health service infrastructure in India has eroded patient trust in formal health services [27]. Trust is one of the most important components for providing effective health care services [27], and lack of such trust can act as an access barrier [28]. In India, the erosion of trust in the formal public health system may have led people to visit IPs, reducing adherence to proven treatment and care, and thus compromising the quality of care [27]. The level of mistrust is greater among the underprivileged in India, who continue to have limited choice and no voice [27].

Many of the respondents using IPs were aware of IPs’ limited qualifications. However, our findings indicate that word of mouth played a larger role than qualifications when choosing service providers. This is unsurprising, as often a familiar or recommended provider is deemed more trustworthy and is thus consulted first for any health care need. Such familiarity is also instrumental in IPs being able to extend credit terms to patients. IPs have also adapted their health care services in line with local peoples’ needs, preferences, and economic power. This is similar to a description of access provided by Penchansky and Thomas [29], who stated ‘the degree of fit between the consumer and the service; the better the fit, the better the access’ [30]. Our findings suggest that IPs develop close relationships with families, and build their reputations among rural communities, making them the preferred choice over formal providers. IPs respect patients’ social contexts and living conditions. The villagers know them well, and their past successes create a perception of trustworthiness and dependability [31].

Most villages are not well connected to urban population centres where qualified doctors practice health care, and access to both public and private health care sectors remains poor. It is critical for UHC to ensure equitable distribution of health services with one of the key levers being filling the physical and infrastructure gap [9]. Although indirect costs such as transportation are not part of UHC, strengthening the public system prioritizing the peoples’ needs is key [3,9]. As the cost of transport is an access driver, local IPs fill the gap. Sometimes, transport costs are more than the fees paid to the FP. Hence, although an indirect cost, transport greatly influences the rural population’s health care choices [10].

The public health community is gradually recognising the IPs’ importance [7] but medical associations in India continue to discourage their inclusion in the formal health care system [32]. Most Indian medical establishments view IPs as dangerous ‘quacks’, and courts have ordered the government to shut down IPs’ operations [33]. These issues need to be resolved if UHC is to be achieved in rural India. A randomised controlled trial training IPs showed a modest rise of 7.9 [CI: 0.4, 15.5] percentage points in correct case management, but little impact on correct medicine prescription [13]. Therefore, complementary interventions including regular monitoring and supervision and changes in financial and non-financial incentives to encourage good practice by IPs could be included. Additional demand-side measures may include public education to reduce information asymmetries between providers and patients and measures to increase awareness of rational drug usage [34]. Affordability, access, and awareness, along with socio-cultural factors and trust need to be addressed by the formal healthcare sector to ensure universal access to care.

### Methodological considerations

We used a multidisciplinary perspective, including public health, social science, and business process management researchers. The FGDs gave detailed insights into how rural patients choose between IPs and FPs. We included participants of different gender and from different societal strata, unfortunately fewer women than men responded to our invitation. Our results’ generalisability is limited to areas with similar conditions. Our study is also limited by not including IPs themselves, which is important in future research.

## Conclusion

Our study found that patients decide their provider based on trust, reputation, and affordability, balancing carefully their symptoms with available providers. To expand access to healthcare in these villages, patients need health care services to offer flexible hours, to be close and readily available, and to combine consultations for diagnoses with the dispensing of medications, as well as decreasing the cost of healthcare or providing health insurance for the village population. It is important to note that improving the public health system for rural populations and achieving universal health coverage calls for more than just tackling the shortage of qualified doctors or FPs, and may include involving the large numbers of trusted informal providers.
